# The epidemiology of subclinical malaria infections in South-East Asia: findings from cross-sectional surveys in Thailand–Myanmar border areas, Cambodia, and Vietnam

**DOI:** 10.1186/s12936-015-0906-x

**Published:** 2015-09-30

**Authors:** Mallika Imwong, Thuy Nhien Nguyen, Rupam Tripura, Tom J. Peto, Sue J. Lee, Khin Maung Lwin, Preyanan Suangkanarat, Atthanee Jeeyapant, Benchawan Vihokhern, Klanarong Wongsaen, Dao Van Hue, Le Thanh Dong, Tam-Uyen Nguyen, Yoel Lubell, Lorenz von Seidlein, Mehul Dhorda, Cholrawee Promnarate, Georges Snounou, Benoit Malleret, Laurent Rénia, Lilly Keereecharoen, Pratap Singhasivanon, Pasathorn Sirithiranont, Jem Chalk, Chea Nguon, Tran Tinh Hien, Nicholas Day, Nicholas J. White, Arjen Dondorp, Francois Nosten

**Affiliations:** Mahidol Oxford Research Unit, Faculty of Tropical Medicine, Mahidol University, Bangkok, Thailand; Department of Molecular Tropical Medicine and Genetics, Faculty of Tropical Medicine, Mahidol University, Bangkok, Thailand; Oxford University Clinical Research Unit, Hospital for Tropical Diseases, 764 Vo Van Kiet, District 5, Ho Chi Minh City, Vietnam; Shoklo Malaria Research Unit, Faculty of Tropical Medicine, Mahidol University, Tak, Thailand; Center for Malariology, Parasitology and Entomology Control, Phan Rang-Thap Cham, Ninh Thuan Province Vietnam; Institute of Malariology-Parasitology, Entomology (IMPE) of Ho Chi Minh City, 699 Tran Hung Dao Q5, Ho Chi Minh City, Vietnam; Centre for Tropical Medicine and Global Health, Nuffield Department of Medicine, Churchill Hospital, Oxford, UK; WWARN Asia Regional Centre, Mahidol University, Bangkok, Thailand; Sorbonne Universités, UPMC Univ Paris 06, UPMC UMRS CR7, 75005 Paris, France; Centre d’Immunologie et de Maladies Infectieuses (CIMI), Paris, Institut National de la Santé et de la Recherche Médicale (Inserm) U1135, Centre National de la Recherche Scientifique (CNRS) ERL 8255, 75013 Paris, France; Singapore Immunology Network (SIgN), Agency for Science, Technology and Research (A*STAR), Biopolis, Singapore, 278177 Singapore; Department of Microbiology, Yong Loo Lin School of Medicine, National University of Singapore, National University Health System, 5 Science Drive 2, Blk MD4, Level 3, Singapore, 117597 Singapore; Department of Tropical Hygiene, Faculty of Tropical Medicine, Mahidol University, Bangkok, Thailand; National Center for Parasitology, Entomology and Malaria Control, No. 372, Preah Monivong, Phnom Penh, 12302 Cambodia; Armed Forces Research Institute of Medical Sciences (AFRIMS), Bangkok, Thailand

**Keywords:** Malaria, *P. falciparum*, *P. vivax*, Sub-microscopic, Epidemiology, South-East Asia, Myanmar, Thailand, Cambodia, Vietnam, Greater Mekong Sub-region

## Abstract

**Background:**

The importance of the submicroscopic reservoir of *Plasmodium* infections for malaria elimination depends on its size, which is generally considered small in low transmission settings. The precise estimation of this reservoir requires more sensitive parasite detection methods. The prevalence of asymptomatic, sub-microscopic malaria was assessed by a sensitive, high blood volume quantitative real-time polymerase chain reaction method in three countries of the Greater Mekong Sub-region.

**Methods:**

Cross-sectional surveys were conducted in three villages in western Cambodia, four villages along the Thailand–Myanmar border and four villages in southwest Vietnam. Malaria parasitaemia was assessed by *Plasmodium falciparum*/pan malaria rapid diagnostic tests (RDTs), microscopy and a high volume ultra-sensitive real-time polymerase chain reaction (HVUSqPCR: limit of detection 22 parasites/mL). All villagers older than 6 months were invited to participate.

**Results:**

A census before the surveys identified 7355 residents in the study villages. Parasite prevalence was 224/5008 (4 %) by RDT, 229/5111 (5 %) by microscopy, and 988/4975 (20 %) when assessed by HVUSqPCR. Of these 164 (3 %) were infected with *P. falciparum*, 357 (7 %) with *Plasmodium vivax*, 56 (1 %) with a mixed infection, and 411 (8 %) had parasite densities that were too low for species identification. A history of fever, male sex, and age of 15 years or older were independently associated with parasitaemia in a multivariate regression model stratified by site.

**Conclusion:**

Light microscopy and RDTs identified only a quarter of all parasitaemic participants. The asymptomatic *Plasmodium* reservoir is considerable, even in low transmission settings. Novel strategies are needed to eliminate this previously under recognized reservoir of malaria transmission.

**Electronic supplementary material:**

The online version of this article (doi:10.1186/s12936-015-0906-x) contains supplementary material, which is available to authorized users.

## Background

Eliminating the submicroscopic reservoir of *Plasmodium* infections in asymptomatic carriers may play a critical role in the elimination of malaria [1]. In low transmission settings, such as in countries of the Greater Mekong Sub-region (GMS), asymptomatic carriage is generally considered to be low. Estimates of the asymptomatic reservoir size largely relying on methods with limited sensitivity have prevented a more complete understanding of the epidemiology of malaria. Light microscopy and rapid diagnostic tests (RDT) have comparable lower limits of detection. Assessment by calibration with spiked samples shows a limit between 10 and 100 parasites/µL (10,000–100,000/mL) for microscopy of a thick blood film [2]. In comparison PCR methods have better sensitivity, typically detecting 5–10 parasites/µL (5000–10,000/mL), although sensitivity depends on the volume of blood examined [3, 4]. For instance, in a filter paper blood spot of 5 µL (0.005 mL), parasite densities lower than 1/5 µL (=200 parasites/ml) are unlikely to be detected irrespective of the sensitivity of the PCR method itself.

A series of cross sectional surveys were conducted along the Thailand–Myanmar border, in Western Cambodia, and Vietnam using a sensitive PCR detection method based on larger blood volumes than conventional PCR methods use [5]. In the context of the threat of artemisinin and multi drug resistant falciparum malaria, countries in the GMS have adopted recently a malaria elimination agenda. The findings of these prevalence studies are critical to target interventions for malaria elimination.

## Methods

**Fig. 1:**
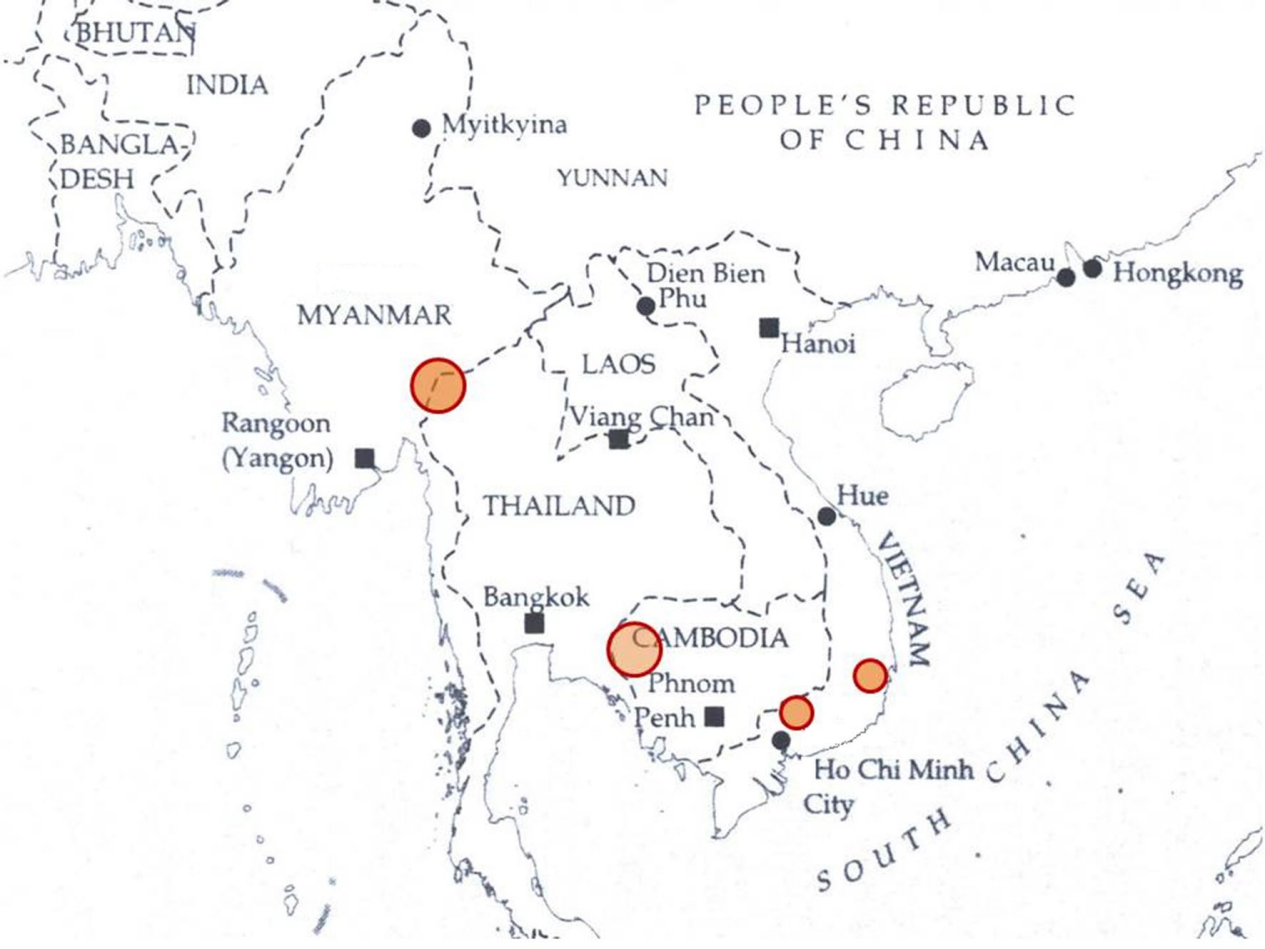
South East Asia, with markers for the position of the study sites in Thailand–Myanmar border areas, Cambodia and two sites in Vietnam

Surveys were performed in malaria-endemic areas along the Thailand–Myanmar border, in western Cambodia, and south-western Vietnam (Fig. 1). In these areas, malaria transmission is low, heterogeneous, and seasonal with entomological inoculation rates generally below one/person/year. The majority of clinical cases occur during the rainy season between May and December [6–9]. *Plasmodium vivax* and *P. falciparum* have historically each comprised approximately half the clinical cases, although with recent reductions in overall malaria incidence, *P. vivax* now predominates [10]. The region has been recognized as the origin of anti-malarial drug resistance in *P. falciparum* to chloroquine, sulfadoxine-pyrimethamine and mefloquine. More recently, *P. falciparum* strains with reduced susceptibility to artemisinins have been detected in this region [11–14].

## Locations

### Thailand–Myanmar border

In response to large numbers of malaria cases in Myanmar close to the North-western border with Thailand and requests for assistance, health posts with facilities for malaria diagnosis and treatment were installed in several villages. These were the focal point for the conduct of limited cross- sectional surveys in 16 villages to plan optimum control interventions (Additional file 1: Table S1). Based on the findings more exhaustive surveys were conducted in four villages (HKT, KNH, TOT, TPN) located within 10 km of the Thailand border considered representative of the area in terms of environment, ecology, population, and behaviour.

### Cambodia

*Plasmodium falciparum* with reduced susceptibility to artesunate monotherapy was first detected in Battambang [15] and then Pailin, Western Cambodia [12]. Many of the containment efforts in Cambodia have been focused on Pailin resulting in a marked decline in clinical malaria incidence over the last few years [16–20]. In 2013, the Cambodian National Malaria Control Programme and Mahidol-Oxford Research Unit formed a malaria research team based in Pailin Referral Hospital to investigate if there are areas with significant subclinical malaria parasitaemia. Three villages (KL, OK, and PDB) were selected based on the highest incidence of clinical falciparum malaria in the village malaria workers’ records from 2012.

### Vietnam

Malaria remains a public health challenge in Vietnam despite a substantial reduction in the incidence of disease over the last 20 years. Since 2010, studies in Binh Phuoc province show an increased proportion of slow clearing artemisinin-resistant infections [11], but still with satisfactory cure rates with ACT (dihydroartemisinin–piperaquine) [21]. Two villages (BK and BB) in Dak O commune of Binh Phuoc and two villages (GIA and THA) in Ninh Thuan province were selected for further evaluation based on surveillance data from pilot studies. The study in Vietnam has been conducted in collaboration with malaria control programme of Vietnam (Institute of Malariology, Parasitology, and Entomology (IMPE) Ho Chi Minh City and IMPE Qui Nhon).

## Procedures

In each village a committee was formed composed of village leaders, village malaria workers, and volunteers. The committees assisted the study team in organizing the survey and in engaging and mobilizing the community. A census was performed before the survey.

During the surveys all individuals aged 6 months or above were invited to participate, including temporary residents and migrant workers. Individual informed consent was obtained from adults, and parental consent for the participation of children under 16 years. No additional assent was obtained from adolescents. Demographic information was collected and the tympanic temperature, weight, and height of all individuals were measured. A brief history of recent illness and travel was also obtained. Venous blood (3 mL) was collected from all individuals aged ≥5 years, and 500 µL from children aged ≥6 months–5 years. Participants with fever ≥37.5 °C were tested for malaria by rapid diagnostic test (RDT), and were treated if positive according to national guidelines.

The blood samples were stored in a cool box in the field and then transported within 12 h to the local laboratory for further sample processing and RDT assessment. In Cambodia, the Healgen malaria *P. falciparum*/Pan one-step RDT was used (Zhejiang Orient Biotech, China); in Thailand–Myanmar border areas and in Vietnam, the SD Bioline Malaria Ag Pf/Pan POCT was used (Cat. No. 05FK60; 65, Borahagal-ro, Giheung-gu, Yongin-si, Gyeonggi-do, Republic of Korea). The RDTs distinguish between *P. falciparum infections*, non-*P. falciparum infections*, and no infections. Haemoglobin was measured using Hemocue (Ängelholm, Sweden). Sample processing for the quantitative PCR included separation of plasma, buffy coat, and packed red blood cells, which were frozen and stored at −80 °C. The frozen samples from the Thailand–Myanmar Border and Cambodia were transported monthly on dry ice to the laboratory in Bangkok, Thailand and the samples from the Vietnam sites were shipped to Ho Chi Minh City, Vietnam for DNA extraction and quantitative PCR.

## Quantification of malaria parasitaemia

Standard microscopy was performed by microscopists who had at least 5 years experience and/or were confirmed to be Level 2 or better with a WHO 55 slide set. The number of parasites/500 white blood cells was counted on Giemsa-stained thick films.

Detailed description, evaluation and validation of the high-volume ultrasensitive real-time polymerase chain reaction (HVUSqPCR) methods have been reported recently in detail [5]. In summary, the DNA template for PCR detection and quantification of *Plasmodium* was purified from the thawed packed red blood cells samples. Purified DNA was dehydrated in a centrifugal vacuum concentrator and then suspended in a small volume of PCR grade water resulting in a concentration factor defined by the original blood volumes (100–2000 μL) divided by the resuspension volume (10–50 µL). Two microlitres of resuspended DNA was used as template in the qPCR reaction. The presence of malaria parasites and an estimate of the parasite numbers (genomes) in each sample were assessed by an absolute quantitative real-time PCR (qPCR) method (Quanti-Tect Multiplex PCR No ROX^®^, QIAGEN, Germany). The 18S rRNA-targeting primers and hydrolysis probes used in the assay have been validated and are highly specific for *Plasmodium* species [22]. The lower limit of accurate quantitation of this method is 22 parasites/ml of whole blood [14].

For samples where the HVUSqPCR was positive, an attempt was made to determine the *Plasmodium* species present using nested PCR protocols specific to *P. falciparum* (microsatellite marker Pk2), *P. vivax* (microsatellite marker 3.502) and *Plasmodium malariae* (18s rRNA) as described previously [22–24]. Samples for which there was insufficient DNA to do this, or where no amplification was obtained in this step were reported as being of indeterminate species (*Plasmodium* spp.).

### Statistical analyses

For the purposes of analysis, fever was defined as a tympanic temperature >37.5 °C. Anaemia was defined as “None” if the haemoglobin (Hb) was ≥11 g/dL, “Mild” if Hb ≥8–<11 g/dL, and “Moderate” if Hb <8 g/dL. Characteristics of the study population and clinical association with presence of parasitaemia were compared using the Chi squared test. Nonparametric testing for trend was performed using the *nptrend* command in STATA which is based on the Wilcoxon rank-sum test. The detection of parasitaemia was summarized by location, age and gender strata as specified a priori in the analysis plan. Sensitivities and specificities for malaria diagnosis were calculated using HVUSqPCR as the reference standard. Overall and study location specific risk factors for parasite carriage (detected by HVUSqPCR) were assessed by logistic regression with household fitted as a random effect. For all models, a stepwise approach was used and only variables significant at the 0.05 level were retained in the final models. All analyses were performed using Stata, version 13 (StataCorp, College Station, TX, USA).

### Ethics approval

The studies were approved by the Cambodian National Ethics Committee for Health Research (0029 NECHR, dated 04 Mar 2013) the Institute of Malariology, Parasitology and Entomology in Ho Chi Minh City (185/HDDD dated 15 May 2013), the Institute of Malariology, Parasitology and Entomology in Qui Nhon (dated 14 Oct 2013) and the Oxford Tropical Research Ethics Committee (1015-13, dated 29 Apr 2013).

## Results

**Fig. 2:**
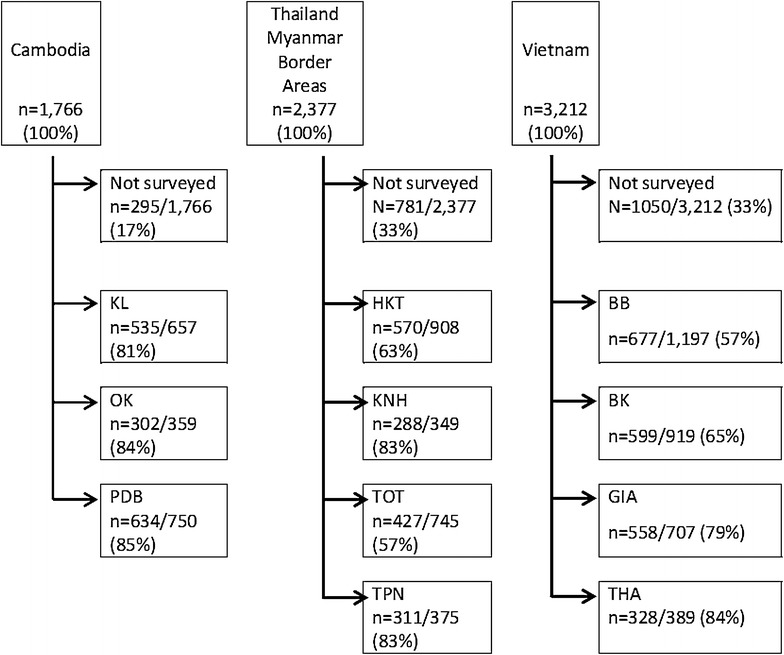
Assembly of study participants

A census prior to the surveys identified 7355 residents in 11 study villages, 1766 in three Cambodian villages (KL, OK, and PDB), 2377 in four villages along the Thailand–Myanmar border (TOT, TPN, KNH, and HKT) and 3212 in four villages in Vietnam (BB, BK, THA, GIA) (Fig. 2). In Cambodia the surveys were conducted in June 2013, along the Thailand–Myanmar border between May and July 2013, and in October and November 2013 in Vietnam. Of the census population, 83 % participated in the surveillance in the Cambodian villages, 67 % in villages along the Thailand–Myanmar border and 67 % in the Vietnamese villages. The most frequently reported reasons for non-participation were problems related to travel and refusal of consent (Additional file 2).

### Characteristics of the study population

**Fig. 3:**
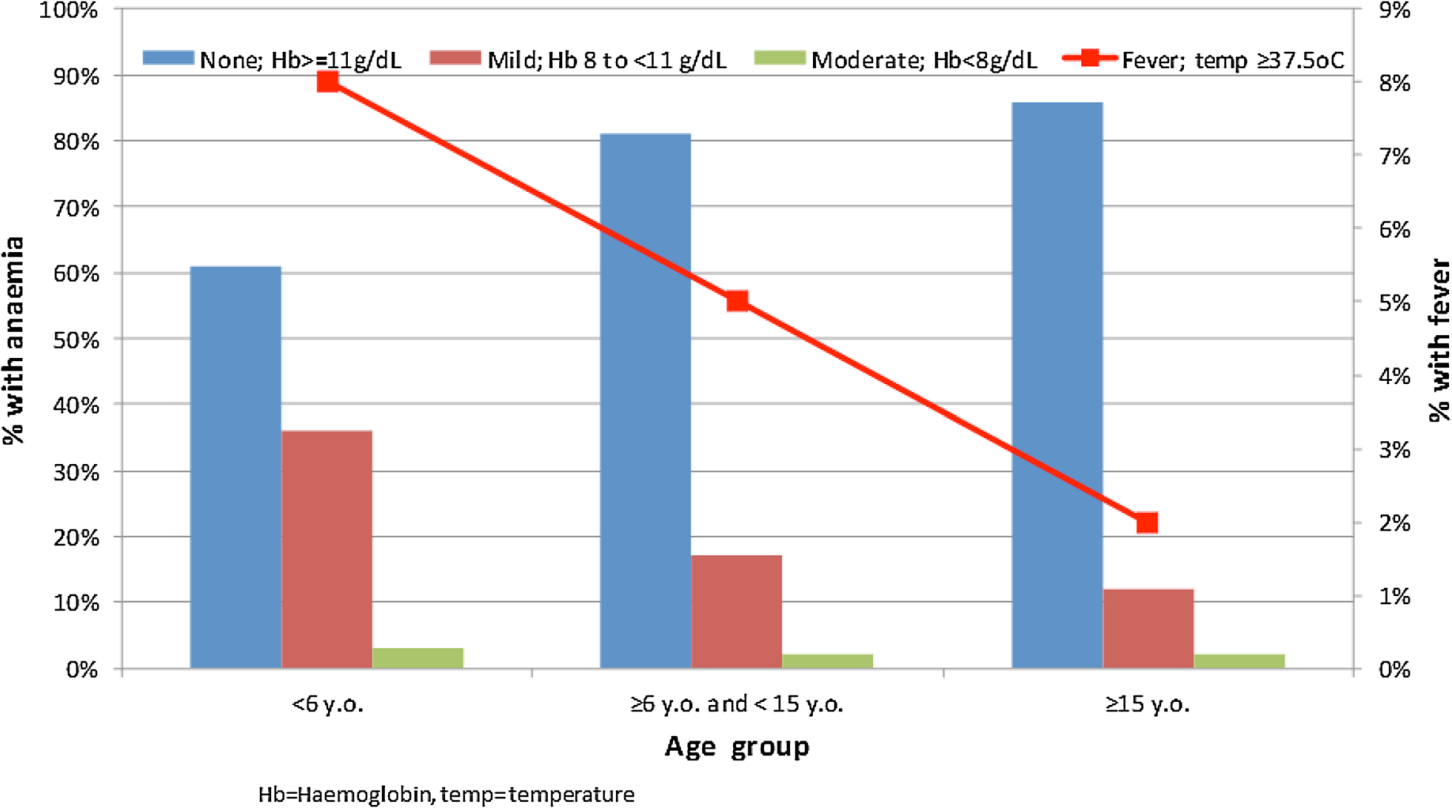
The correlations between anaemia, fever and age. Younger participants were more likely to be febrile and anaemic (Hb < 11 g/dL)

The population of the 11 participating villages were comparable in age distributions; the median age of the participants was 21 years with 37 % of the participants under 15-year-old (Table 1; data disaggregated by village are shown in Additional file 3). Of the 741 children under 72 months who participated in the study 57 (8 %) were febrile (tympanic temperature >37.5 °C) on the day of the examination. In the Vietnamese sites the proportion of febrile children was 1 % in contrast to 10 % in the Thailand–Myanmar border areas and the Cambodian sites (p = 0.001; Table 1). Moderate anaemia (Hb <8 g/dL) was present in 5 % of children in the Vietnamese sites, 3 % of children in the Thailand–Myanmar border areas and 2 % in the Cambodian sites (p < 0.009). Younger age was associated with a higher prevalence of fever and anaemia compared to older age (p < 0.0001; Fig. 3). The prevalence of fever increased with the severity of anaemia: 3 % in participants with an Hb ≥11 g/dL, 5 % with 8 ≤ Hb < 11 g/dL and 9 % in the group with Hb <8 g/dL (2 d.f.; p = 0.002).

**Table 1:** Study population characteristics in the malaria surveys

	Cambodia	Thai–Myanmar border areas	Vietnam	Overall
Median age, years (IQR, range)	21 (9–35, 0.3–83)	20 (9–37, 0.2–94)	22 (10–36, 0.1–94)	21 (9–36, 0.1–94)
Children <15 y.o. (%)	660/1766 (37 %)	923/2373 (39 %)	1112/3194 (35 %)	2695/7333 (37 %)
Male (%)	901/1766 (51 %)	1265/2377 (53 %)	1619/3207 (50 %)	3785/7350 (51 %)
Children <72 m.o. and febrile (%)^a^	25/242 (10 %)	30/315 (10 %)	2/184 (1 %)	57/741 (8 %)
No anaemia (Hb ≥11 g/dL)	1243 (86 %)	1319 (87 %)	1535 (74 %)	4097 (81 %)
Mild anaemia (Hb 8–<11 g/dL)	188 (13 %)	182 (12 %)	445 (21 %)	815 (16 %)
Moderate anaemia (Hb <8 g/dL)	15 (1 %)	18 (1 %)	91 (4 %)	124 (2 %)

*IQR* inter-quartile range, *y.o.* years old, *m.o.* months old, *g/dL* grams per decilitre

^a^Tympanic temperature >37.5 °C

### Detection of *Plasmodium* parasitaemia

Using rapid diagnostic tests (RDT) overall 224/5008 (4 %) participants tested positive (Table 2). Only 1/1447 participants in the Cambodian sites tested positive compared with 158/1384 (11 %) along the Thailand–Myanmar border and 65 of 2177 (3 %) in Vietnam. With microscopy 229/5111 (5 %) showed *Plasmodium* parasites; 1 % in Cambodia, 4 % in Vietnam and 9 % on the Thailand–Myanmar border. Using HVUSqPCR 988/4975 (20 %) participants’ blood samples tested positive for the presence of *Plasmodium* DNA, of which 164/4975 (3 %) were *P. falciparum*, 357 (7 %) *P. vivax*, 56 (1 %) were mixed infections and in 411 (8 %) of specimens it was not possible to determine the species because of the low parasite DNA content. The data disaggregated by village are shown in Additional file 4. No *Plasmodium* species other than *P. falciparum* and *P. vivax* were detected.

**Table 2:** The results of RDT, light microscopy, and HVUSqPCR in Cambodia, Thailand–Myanmar border areas, and Vietnam

	Cambodia	Thailand–Myanmar border areas	Vietnam	Overall
RDT				
n	1447	1384	2177	5008
No. pos	1 (0.1 %)	158 (11 %)	65 (3 %)	224 (5 %)
Pf	1 (0.1 %)	108 (8 %)	39 (2 %)	148 (3 %)
Non-PF	0	50 (4 %)	18 (1 %)	68 (1 %)
Mixed	0	0	8 (0.4 %)	8 (0.2 %)
Microscopy^a^				
n	1447	1532	2132	5111
No. pos	8 (1 %)	144 (9 %)	77 (4 %)	229 (5 %)
Pf	1 (0.1 %)	39 (3 %)	27 (1 %)	67 (1 %)
Pv	7 (0.5 %)	105 (7 %)	46 (2 %)	158 (3 %)
HVUSqPCR				
n	1447	1536	1992	4975
No. pos	229 (16 %)	520 (34 %)	239 (12 %)	988 (20 %)
Pf	32 (2 %)	87 (6 %)	45 (2 %)	164 (3 %)
Pv	48 (3 %)	230 (15 %)	79 (4 %)	357 (7 %)
Mixed	4 (0.3 %)	21 (1 %)	31 (2 %)	56 (1 %)
*P*. spp.	145 (10 %)	182 (12 %)	84 (4 %)	411 (8 %)

N, number; No. pos, number positive; mixed, mixed infections *P. falciparum* and *P. vivax*; Pf, *P. falciparum*; non-PF, *Plasmodium* species other than *P. falciparum*; Pv *P. vivax*

^a^Light microscopy did not detect mixed infections

**Fig. 4:**
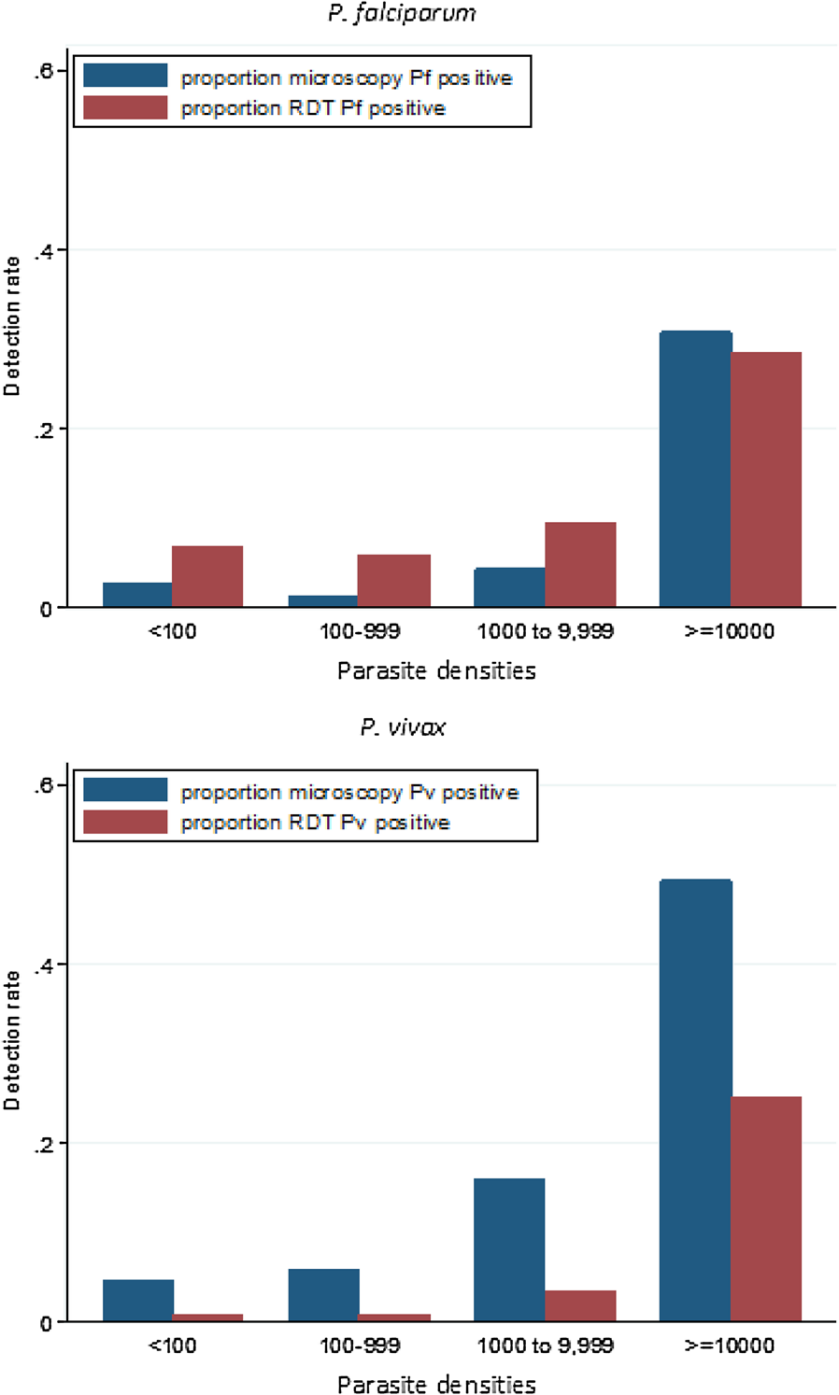
The detection rate of microscopy and RDT in relation to parasite density

Using HVUSqPCR as the reference-standard, the overall sensitivity for RDTs to detect *P. falciparum* infections was 44 % with a specificity of 99 % (Table 3). The sensitivity of RDTs for detecting non-*P. falciparum* infections was only 14 % with a specificity of 100 %. Sensitivity of microscopy compared to HVUSqPCR was similar for *P. falciparum* (30 %) and for *P. vivax* (35 %; Table 4; the disaggregated data are shown in Additional file 5). The sensitivity of both RDTs (range 0–68 %) and microscopy (range 0–40 %) for detecting asymptomatic malaria was variable between sites, whereas specificity for both RDTs (range 97–100 %) and microscopy (100 %) was high in all sites. Sensitivity of RDTs and microscopy was better at higher parasite densities (Fig. 4). In the very low parasitaemias in which species could not be determined by PCR (*Plasmodium* spp.), the sensitivity of RDTs was 5 % and of microscopy 3 %.

**Table 3:** Species specific sensitivity, specificity, positive predictive value and negative predictive value of RDTs compared with HVUSqPCR parasite detection

	qPCR pos, RDT pos	qPCR pos, RDT neg	qPCR neg, RDT neg	qPCR neg, RDT pos	Total	Sensitivity (95 % CI)	Specificity (95 % CI)	Positive predictive value (95 % CI)	Negative predictive value (95 % CI)
Pf									
All sites	96	120	4462	55	4733	44 % (38–51 %)	99 % (98–99 %)	64 % (55–71 %)	97 % (97–98 %)
Cam	1	35	1411	0	1447	3 % (0–15 %)	100 % (99–100 %)	100 % (3–100 %)	98 % (97–98 %)
TMBA	71	34	1241	37	1383	68 % (58–76 %)	97 % (96–98 %)	66 % (56–75 %)	97 % (96–98 %)
Viet	24	51	1810	18	1903	32 % (22–44 %)	99 % (98–100 %)	57 % (41–72 %)	97 % (96–98 %)
Non Pf									
All sites	54	332	4332	15	4733	14 % (11–18 %)	100 % (99–100 %)	78 % (67–87 %)	93 % (92–94 %)
Cam	0	52	1395	0	1447	0 % (0–1 %)	100 % (93–100 %)	NA	96 % (95–97 %)
TMBA	47	180	1153	3	1383	21 % (16–27 %)	100 % (99–100 %)	94 % (84–99 %)	87 % (85–88 %)
Viet	7	100	1784	12	1903	7 % (3–13 %)	100 % (99–100 %)	37 % (16–62 %)	95 % (94–96 %)
*P*. spp.									
All sites	20	379	3740	46	4185	5 % (3–8 %)	99 % (98–99 %)	30 % (20–43 %)	91 % (90–92 %)
Cam	0	145	1218	0	1363	0 % (0–0.3 %)	100 % (97–100 %)	NA	89 % (88–91 %)
TMBA	16	158	873	24	1071	9 % (5–15 %)	97 % (96–98 %)	40 % (25–57 %)	85 % (82–87 %)
Viet	4	76	1649	22	1751	5 % (1–12 %)	99 % (98–99 %)	15 % (4–35 %)	96 % (95–97 %)

RDT, rapid diagnostic test, HVUSqPCR, high volume ultra-sensitive real time polymerase chain reaction; Pf’, *Plasmodium falciparum* or *Plasmodium falciparum* mixed infection; non-Pf, RDTs distinguish between *P. falciparum*, and non-*P. falciparum*, and uninfected blood. In this study only *P. vivax* was identified by molecular methods so non-*P. falciparum* in an RDT equates with *P. vivax*; *P*. spp., *Plasmodium* species not identified by HVUSqPCR compared against any *Plasmodium* infection detected by RDT; Cam, Cambodia; TMBA, Thailand–Myanmar border areas; qPCR pos, positive (species specific); qPCR neg, negative; NA, not applicable

**Table 4:** Species specific sensitivity, specificity, positive predictive value and negative predictive value of microscopy compared with HVUSqPCR parasite detection

	qPCR pos micro pos	qPCR pos micro neg	qPCR neg micro neg	qPCR neg micro pos	Total	Sensitivity (95 % CI)	Specificity (95 % CI)	Positive predictive value (95 % CI)	Negative predictive value (95 % CI)
Pf									
All sites	62	144	4616	5	4827	30 % (24–37 %)	100 % (99–100 %)	93 % (83–98 %)	97 % (96–97 %)
Cam	0	36	1410	1	1447	0 % (0–10 %)	100 % (99–100 %)	0 % (0–10 %)	98 % (97–98 %)
TMBA	37	61	1418	1	1517	38 % (28–48 %)	100 % (99–100 %)	98 % (86–100 %)	96 % (95–97 %)
Viet	25	47	1788	3	1863	35 % (24–47 %)	100 % (99–100 %)	89 % (72–98 %)	97 % (97–98 %)
Pv									
All sites	141	262	4411	13	4827	35 % (30–40 %)	100 % (99–100 %)	92 % (86–95 %)	94 % (94–95 %)
Cam	7	45	1395	0	1447	14 % (6–26 %)	100 % (99–100 %)	100 % (59–100 %)	97 % (96–98 %)
TMBA	99	149	1264	5	1517	40 % (34–46 %)	100 % (99–100 %)	95 % (89–98 %	89 % (89–91 %)
Viet	35	68	1752	8	1863	34 % (25–44 %)	100 % (99–100 %)	81 % (67–92 %)	96 % (95–97 %)
*P*. spp									
All sites	13	391	3864	1	4269	3 % (2–5 %)	100 % (99–100 %)	93 % (66–100 %)	91 % (90–92 %)
Cam	0	145	1218	0	1363	0 % (0–0.3 %)	100 % (97–100 %)	NA	89 % (88–91 %)
TMBA	6	175	1009	0	1190	3 % (1–7 %)	100 % (99–100 %)	100 % (54–100 %)	85 % (83–87 %)
Viet	7	71	1637	1	1716	9 % (4–18 %)	100 % (99–100 %)	88 % (47–100 %)	96 % (95–97 %)

HVUSqPCR, high volume ultra-sensitive real time polymerase chain reaction; Pf, *Plasmodium falciparum* or *Plasmodium falciparum* mixed infection; Pv, *Plasmodium vivax*; *P*. spp., *Plasmodium* species not identified by HVUSqPCR compared against any *Plasmodium* infection detected by RDT; Cam, Cambodia; TMBA, Thailand–Myanmar border areas; qPCR pos, positive (species specific); qPCR neg, negative; NA, not applicable

### Epidemiological and clinical associations with *Plasmodium* infections

**Fig. 5:**
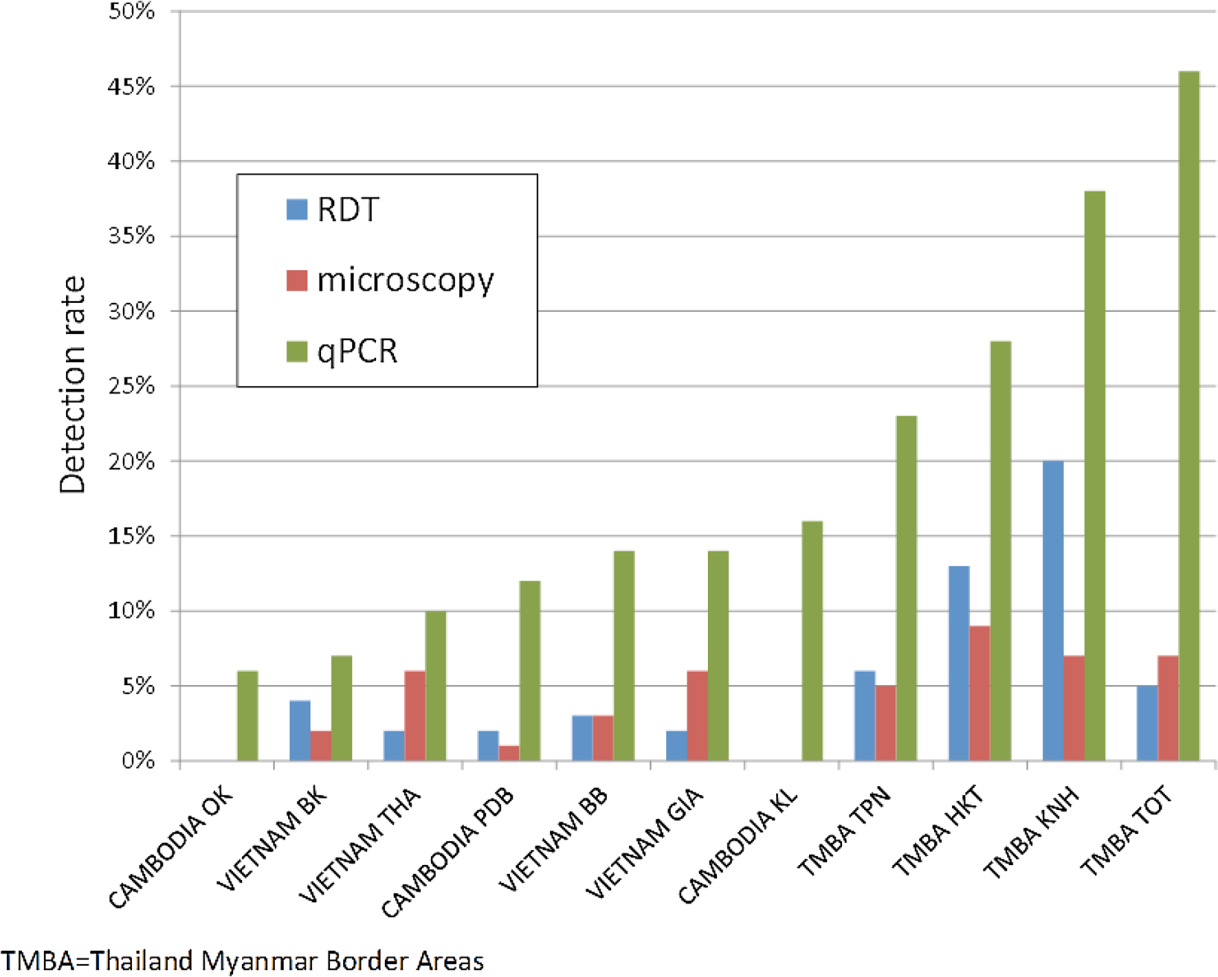
Prevalence summary by detection method for each study village (sorted by high volume ultra-sensitive real time polymerase chain reaction; HVUSqPCR)

Parasite prevalence detected by HVUSqPCR was lowest in Vietnam (239/1992; 12 %), followed by Cambodia (16 %; 229/1447) and (34 %; 520/1536) on the Thailand–Myanmar border (p < 0.0001; Fig. 5). Parasitaemia was detected in all age groups and prevalence increased with age (Additional file 6). In every village parasite prevalence was higher in males (592/2462; 24 %) compared to females (396/2509; 16 %; p < 0.0001).

**Fig. 6:**
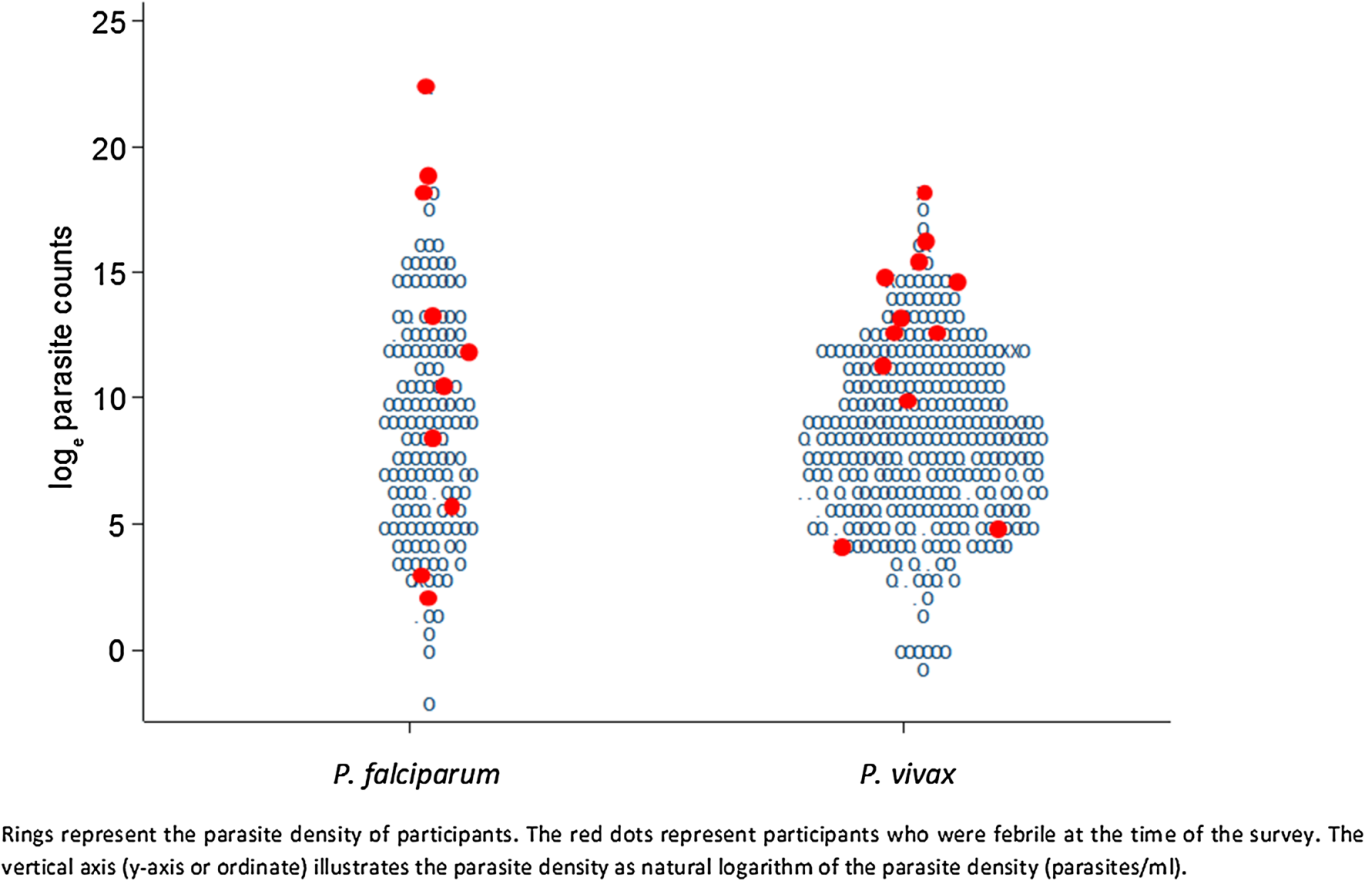
Fever in relation to *P. falciparum* and *P. vivax* densities

A total of 10/152 (7 %) participants with *P. falciparum* infections detected by HVUSqPCR were febrile on the day of the survey in contrast to 14/323 (4 %) with *P. vivax* infections, 112/3412 (3 %) without parasitaemia and 0/48 with mixed infections (comparison *P. falciparum* and negatives: p = 0.038; comparison *P. vivax* and negatives p = 0.3 and comparison *P. falciparum* and *P. vivax* p = 0.4). The geometric mean (95 %CI) parasite density in the 47 febrile patients was 3729 (755–18,420) parasites/mL, compared to 1151 (905–1464) parasites/mL in afebrile individuals. For both *P. falciparum* or *P. vivax* was it impossible to determine a clearly delineated pyrogenic parasite density threshold (Fig. 6).

In participants carrying *P. falciparum*, mild anaemia (Hb 8–<11 g/dL) was present in 30/163 (18 %) participants and moderate anaemia (Hb <8 g/dL) in 7/163 (4 %) (Table 5). Among the participants with *P. vivax* 39/354 (11 %) had mild anaemia and 5/354 (1 %) had moderate anaemia (for comparison mild anaemia between Pf and Pv p = 0.026; comparison moderate anaemia p = 0.058). Among 3889 participants without evidence of parasitaemia by any detection method 619 (16 %) had mild anaemia and 96 (2 %) had moderate anaemia. Parasitaemic participants without anaemia (n = 819) had a geometric mean (95 % CI) parasite density of 1159 (912–1474) parasites/mL, compared to 608 (295–1253) parasites/ml in those with mild anaemia (n = 137), and 1787 (115–27,757) parasites/ml with moderate anaemia. Parasitaemic participants who were febrile on the day of the survey were more likely to have mild anaemia (11/45; 24 %) or moderate anaemia (4/45; 9 %) compared to afebrile participants (117/841; 14 %; p = 0.05 and 11/841; 1 %; p = 0.005, respectively).

**Table 5:** Mild and moderate anaemia in relation to *P. falciparum* and *P. vivax* infections

HVUSqPCR results	No anaemia; Hb ≥11 g/dL		Mild Hb 8–<11 g/dL		Moderate Hb <8 g/dL		Total
Negative^a^	3174	82 %	619	16 %	96	2 %	3889
Pf	126	77 %	30	18 %	7	4 %	163
Mixed	45	85 %	7	13 %	1	2 %	53
*Plasmodium* spp.^b^	338	84 %	61	15 %	4	1 %	403
Pv	310	88 %	39	11 %	5	1 %	354
Not done	104	60 %	59	34 %	11	6 %	174
Total	4097	81 %	815	16 %	124	2 %	5036

HVUSqPCR, high volume ultra-sensitive real time polymerase chain reaction; Hb, haemoglobin; g/dL, grams per decilitre; Pf, *P. falciparum*, *mixed* more than one *Plasmodium* species identified; *Plasmodium* spp., *Plasmodium* species was not identified; Pv, *P. vivax*

^a^Negative = participants without evidence of parasitaemia by HVUSqPCR

^b^HVUSqPCR assay could not be performed because sample size was not sufficient or other technical reasons

A multivariate logistic regression model was constructed to identify risk factors for parasite carriage (as detected by HVUSqPCR), which included anaemia, fever at the time of the survey, a history of fever, sex, occupation, and age group. Of these, only a history of fever, male sex, and age equal or older 15 years were independently and significantly associated with parasitaemia (Table 6). Country specific models identified in addition an independent association with a past history of malaria in Cambodia and Vietnam (Additional file 7). The models did not suggest different risk factors for a low versus a high density parasitaemia.

**Table 6:** Adjusted odds ratios for being HVUSqPCR positive (n = 967), stratified by site, using random effects modelling (n total = 4807)

Factor	No. HVUSqPCR positive with factor (%)	No. HVUSqPCR positive without factor (%)	Adj. odds ratio	95 % CI
History of fever	105/356 (30 %)	862/4451 (19 %)	1.98	1.54, 2.56
Male	577/2373 (24 %)	390/2434 (16 %)	1.71	1.47, 1.98
Age ≥15^b^	651/3065 (21 %)	Comparator^b^	1.69	1.34, 2.13

Initial model included anaemia (mild, moderate, none), fever at the time of the survey^a^ (0/1), history of fever (0/1), sex, occupation (0/1), family member (0/1) and age group (<6-month-old, 6-year-old–<15-year-old, and ≥15-year-old)

^a^Temperature >37.5 °C

^b^Compared against age <6-year-old [113/616 (18 %)]; age 6-year-old–<15-year-old not significant [203/1126 (18 %), p = 0.11]

## Discussion

This study demonstrates that in areas of the Greater Mekong Subregion (GMS) classified as hypoendemic, a considerable proportion of asymptomatic individuals carry *Plasmodium* parasites. In the GMS and in epidemiologically similar areas in South-East Asia and perhaps beyond the prevalence of malaria infections seems to have been substantially underestimated. The HVUSqPCR *Plasmodium * prevalence estimates in 11 villages in the GMS were approximately four times higher than estimates based on microscopy or RDT. The majority of asymptomatic carriers had *Plasmodium *densities below the lower limits of detection for microscopy, as well as for conventional low volume PCR methods (around 1000–5000 parasites/mL, compared to 22 parasites/mL with HVUSqPCR). Parasite DNA detected by HVUSqPCR is likely to represent living parasites, since mRNA coding for *Plasmodium* species is also detectable in the same patient samples (Dr. Z. Bozdech, personal communication). The findings suggest that the submicroscopic parasite reservoir could be important for transmission between seasons. It will be important to study the longevity and transmissibility of these infections within the human host.

In this study, molecular methods were not used to assess gametocytaemia which would require RNA measurement. This represents a limitation of the study since light microscopy which was used has a much more limited sensitivity and did not detect gametocytaemia [25]. Submicroscopic levels of gametocytaemia, although less efficient, can still transmit malaria [26]. Single point prevalence assessments using a much more sensitive mRNA method may still only have a limited predictive value, since parasitaemia (and presumably gametocytaemia) fluctuates over time and gametocytes may accumulate in the dermis. Longitudinal studies to assess asexual and sexual stage carriage over time using molecular techniques are underway.

There was substantial heterogeneity between villages in the ratios between RDT, microscopy and HVUSqPCR malaria prevalence rates, which suggests that extrapolation of prevalence from detection rates based on the conventional techniques will be imprecise. The HVUSqPCR technique used in this study has a lower limit of detection of around 100,000 parasites in the entire blood volume of an adult and so will still be unable to detect lower circulating parasitaemias or sequestered non-circulating parasites [27]. Very low level parasitaemias can reflect chronic infections in persons with partial immunity or inadequately treated primary infections [28–31].

Parasite densities were lower in the selected villages in Vietnam and Cambodia compared to the Thailand–Myanmar border areas. In the Cambodian study villages all detected parasitaemias were on or below the threshold of parasite densities detectable by microscopy. In assessing these very low parasite densities, false positive PCR results are a concern. A rigorous quality control system was in place throughout the study, and the consistent HVUSqPCR negativity of control samples provide reassurance that the prevalence estimates are not inflated.

In 38 specimens, RDTs detected *P. falciparum* and in eight specimens *P. vivax* parasites while the HVUSqPCR was negative. In the *PfHRP2*-based RDTs for *P. falciparum* this could be due to persistent antigen after the infection has been eliminated. *PfHRP2* is eliminated slowly and thus could accumulate in chronic infections. Alternatively this finding could reflect true false positive RDTs. In 411/988 (42 %) of infections detected by HVUSqPCR, it was not possible to determine the *Plasmodium* species as there was insufficient *Plasmodium* DNA to perform the species identifying nested PCRs. Even more sensitive techniques will be needed to determine whether the species composition of these “undetermined infections” is similar to the composition of the identified species.

## Conclusions

The findings of this study have implications for control, elimination, and eradication of malaria, and in particular for the urgent need to contain and eliminate artemisinin resistant falciparum malaria in South-East Asia. Screening and treatment activities will only identify a minority of parasitaemic individuals, and will not eliminate malaria rapidly enough to stop the spread of artemisinin and multidrug resistant malaria [32]. The only way to eliminate malaria rapidly in populations with significant subpatent malaria prevalence is to treat the entire population with effective anti-malarial drugs. How, when and how often this should be done needs urgent further exploration.

## Additional files

10.1186/s12936-015-0906-x-S1.docx Parasite prevalence in 12 villages along the Thailand–Myanmar border. The villages are sorted by parasite prevalence. The four villages with the highest prevalence were selected for more exhaustive studies.
10.1186/s12936-015-0906-x-S2.docx Reasons why individuals were not included in the malaria surveys.
10.1186/s12936-015-0906-x-S3.docx Study population characteristics in the malaria surveys (disaggregated by village).
10.1186/s12936-015-0906-x-S4.docx The results of RDT, light microscopy, and HVUSqPCR in Cambodia, Thailand Myanmar border areas, and Vietnam (disaggregated by village).
10.1186/s12936-015-0906-x-S5.docx Sensitivity, specificity, positive predictive value and negative predictive value of RDTs and microscopy compared with HVUSqPCR species detection (disaggregated by village).
10.1186/s12936-015-0906-x-S6.tif Parasite prevalence detected by HVUSqPCR by age group.
10.1186/s12936-015-0906-x-S7.docx Adjusted odds for being HVUSqPCR positive stratified by household, random effects modelling by country. In Cambodia the initial model included resident (0/1), anemia (mild, moderate, none), fever (defined as a temperature >37.5 °C) at presentation* (0/1), history of fever (0/1), sex, age group (<6 year-old, ≥6 year-old–<15 year-old, and ≥15 year-old), height, weight, history of illness (0/1), history of malaria (0/1), previous anti-malarials (0/1), use of bednets (0/1), recent visit to forest (0/1), occupation (0/1), family (0/1) and village. In TMBA the initial model included anemia (mild, moderate, none), fever at presentation (0/1), history of fever (0/1), sex, age group <6 year-old, ≥6 year-old–<15 year-old, and ≥15 year-old), occupation (0/1), family (0/1) and village. In Vietnam the initial model included resident (0/1), anemia (mild, moderate, none), fever at presentation* (0/1), history of fever (0/1), sex, age group (<6 year-old, ≥6 year-old–<15 year-old, and ≥15 year-old), height, weight, history of illness (0/1), history of malaria (0/1), use of bednets (0/1), recent visit to forest (0/1), occupation (0/1) and village.
