# Supplementary material for: The epidemiology of subclinical malaria infections in South-East Asia: findings from cross-sectional surveys in Thailand–Myanmar border areas, Cambodia, and Vietnam
Source: Malar J. 2015 Sep 30;14:381. doi: 10.1186/s12936-015-0906-x (PMC4590703; doi:10.1186/s12936-015-0906-x)
Supplement: Supplementary file 1 — 10.1186/s12936-015-0906-x-S1.docx Parasite prevalence in 12 villages along the Thailand–Myanmar border. The villages are sorted by parasite prevalence. The four villages with the highest prevalence were selected for more exhaustive studies. [file 12936_2015_906_MOESM1_ESM.docx]

**Table S1:** Parasite prevalence in **12 villages** along the Thailand-Myanmar border. The villages are sorted by parasite prevalence. The 4 villages with the highest prevalence were selected for more exhaustive studies.

| Village | *P. falciparum* | % | *P. vivax* | % | mix *P. falciparum* + *P. vivax* | % | *Plasmodium* spp | % | negative | % | Total |
| --- | --- | --- | --- | --- | --- | --- | --- | --- | --- | --- | --- |
| KNH | 6 | 12% | 11 | 22% | 2 | 4% | 3 | 6% | 29 | 57% | 51 |
| HKT | 7 | 15% | 9 | 19% | 0 | 0% | 4 | 8% | 28 | 58% | 48 |
| TOT | 3 | 7% | 10 | 24% | 0 | 0% | 4 | 10% | 24 | 59% | 41 |
| TPN | 1 | 2% | 4 | 7% | 1 | 2% | 11 | 20% | 37 | 69% | 54 |
| MPK | 1 | 2% | 13 | 26% | 0 | 0% | 1 | 2% | 35 | 70% | 50 |
| TKY | 1 | 2% | 3 | 5% | 1 | 2% | 7 | 13% | 44 | 79% | 56 |
| KAG | 0 | 0% | 5 | 10% | 0 | 0% | 4 | 8% | 40 | 82% | 49 |
| MWK | 0 | 0% | 4 | 8% | 0 | 0% | 5 | 10% | 41 | 82% | 50 |
| LGA | 0 | 0% | 5 | 10% | 0 | 0% | 3 | 6% | 42 | 84% | 50 |
| HLA | 1 | 2% | 2 | 4% | 1 | 2% | 1 | 2% | 50 | 91% | 55 |
| WMH | 0 | 0% | 4 | 8% | 0 | 0% | 0 | 0% | 47 | 92% | 51 |
| PLL | 1 | 2% | 3 | 5% | 0 | 0% | 0 | 0% | 56 | 93% | 60 |
| TOTAL | 21 | 3% | 73 | 12% | 5 | 1% | 43 | 7% | 473 | 77% | 615 |
