# Supplementary material for: The epidemiology of subclinical malaria infections in South-East Asia: findings from cross-sectional surveys in Thailand–Myanmar border areas, Cambodia, and Vietnam
Source: Malar J. 2015 Sep 30;14:381. doi: 10.1186/s12936-015-0906-x (PMC4590703; doi:10.1186/s12936-015-0906-x)
Supplement: Supplementary file 2 — 10.1186/s12936-015-0906-x-S2.docx Reasons why individuals were not included in the malaria surveys. [file 12936_2015_906_MOESM2_ESM.docx]

**Reasons why individuals were not included in the malaria surveys.**

| Site | Village | No. not surveyed | (%) | Away from village^1^ | (%) | Refused consent | (%) | Busy^2^ | (%) | Died | (%) | Unable^3^ | (%) | Other or Don’t know^4^ | (%) |
| --- | --- | --- | --- | --- | --- | --- | --- | --- | --- | --- | --- | --- | --- | --- | --- |
| Cambodia | KL | 122 | 19% | 85 | 70% | 28 | 23% | 5 | 4% | 2 | 2% | 2 | 2% | 0 | 0% |
|  | OK | 57 | 16% | 50 | 88% | 4 | 7% | 1 | 2% | 0 | 0% | 2 | 4% | 0 | 0% |
|  | PDB | 116 | 16% | 84 | 72% | 14 | 12% | 13 | 11% | 1 | 1% | 4 | 3% | 0 | 0% |
|  | All Cambodia | 295 | 17% | 219 | 74% | 46 | 16% | 19 | 6% | 3 | 1% | 8 | 3% | 0 | 0% |
| Thai Myanmar border | HKT | 337 | 37% | 58 | 17% | 0 | 0% | 107 | 32% | 0 | 0% | 4 | 1% | 168 | 50% |
|  | KNH | 59 | 18% | 26 | 44% | 4 | 7% | 24 | 41% | 0 | 0% | 0 | 0% | 5 | 8% |
|  | TOT | 317 | 43% | 78 | 25% | 0 | 0% | 123 | 39% | 2 | 1% | 2 | 1% | 107 | 34% |
|  | TPN | 61 | 17% | 44 | 72% | 0 | 0% | 1 | 2% | 0 | 0% | 4 | 7% | 12 | 20% |
|  | All Thai Myanmar border | 774 | 33% | 206 | 27% | 4 | 1% | 255 | 33% | 2 | 0% | 10 | 1% | 292 | 38% |
| Vietnam | BB | 476 | 42% | 111 | 23% | 304 | 64% | 0 | 0% | 0 | 0% | 0 | 0% | 61 | 13% |
|  | BK | 237 | 26% | 62 | 26% | 124 | 52% | 0 | 0% | 0 | 0% | 9 | 4% | 42 | 18% |
|  | GIA | 143 | 21% | 0 | 0% | 0 | 0% | 0 | 0% | 0 | 0% | 0 | 0% | 143 | 100% |
|  | THA | 58 | 17% | 0 | 0% | 0 | 0% | 0 | 0% | 0 | 0% | 0 | 0% | 58 | 100% |
|  | All Vietnam | 988 | 31% | 173 | 18% | 428 | 43% | 0 | 0% | 0 | 0% | 9 | 1% | 304 | 31% |
|  | OVERALL | 2057 | 28% | 598 | 29% | 478 | 23% | 274 | 13% | 5 | 0% | 27 | 1% | 596 | 29% |

^1^*Away from village = "not stay in village", "on traveling", “BKK”, “jungle”, “stay at other place”*

^2^*Busy = "farm", "student", “soldier”*

^3^*Unable = Under 6 months, disabled, unable to contact, chronic disease*

^4^*Other/don’t know = “Stay at home”, “unknown”, “move”, and pregnancy*
