# Supplementary material for: The epidemiology of subclinical malaria infections in South-East Asia: findings from cross-sectional surveys in Thailand–Myanmar border areas, Cambodia, and Vietnam
Source: Malar J. 2015 Sep 30;14:381. doi: 10.1186/s12936-015-0906-x (PMC4590703; doi:10.1186/s12936-015-0906-x)
Supplement: Supplementary file 3 — 10.1186/s12936-015-0906-x-S3.docx Study population characteristics in the malaria surveys (disaggregated by village). [file 12936_2015_906_MOESM3_ESM.docx]

Study population characteristics in the malaria surveys. (disaggregated by village)

| Site | Village | Median age (IQR, range), years | Number children (%) less than 15 y.o. | No. (%) male | No. children  <72 months  and febrile (%)* | Anaemia n (%) | | |
| --- | --- | --- | --- | --- | --- | --- | --- | --- |
|  |  |  |  |  |  | None  >=11g/dL | Mild  8 to <11 g/dL | Moderate <8g/dL |
| Cambodia | KL | 21  (9-34, 0.3-83) | 239/657 (36%) | 328/657 (50%) | 10/89 (11%) | 460  (87%) | 63  (12%) | 6  (1%) |
|  | OK | 21  (9-37, 0.9-74) | 135/359 (38%) | 188/359 (52%) | 3/44 (7%) | 249  (84%) | 45  (15%) | 4  (1%) |
|  | PDB | 21  (9-36, 0.5-79) | 286/750 (38%) | 385/750 (51%) | 12/109 (11%) | 534  (86%) | 80  (13%) | 5  (1%) |
|  | All Cambodia | 21  (9-35, 0.3-83) | 660/1,766 (37%) | 901/1,766 (51%) | 25/242 (10%) | 1,243 (86%) | 188 (13%) | 15  (1%) |
| Thailand Myanmar border areas | HKT | 20  (9-37, 0.2-94) | 354/904 (39%) | 480/908 (53%) | 5/100 (5%) | 448  (84%) | 72  (14%) | 11  (2%) |
|  | KNH | 24  (9-40, 0.4-75) | 122/349 (35%) | 190/349 (54%) | 0/49 (0%) | 237  (86%) | 37  (13%) | 3  (1%) |
|  | TOT | 19  (8-35, 0.2-80) | 301/745 (40%) | 384/745 (52%) | 9/103 (9%) | 358  (89%) | 43  (11%) | 3  (1%) |
|  | TPN | 21  (7-37, 0.6-68) | 146/375 (39%) | 211/375 (56%) | 16/63 (25%) | 276  (90%) | 30  (10%) | 1  (0%) |
|  | All Thai-Myanmar border | 20  (9-37, 0.2-94) | 923/2,373 (39%) | 1265/2,377 (53%) | 30/315 (10%) | 1,319 (87%) | 182 (12%) | 18  (1%) |
| Vietnam | BB | 20  (10-35, 0.3-84) | 430/1,184 (36%) | 608/1193 (51%) | 0/12 (0%) | 553  (82%) | 107 (16%) | 13  (2%) |
|  | BK | 22  (10-37, 0.1-79) | 322/916 (35%) | 474/918 (52%) | 1/72 (1%) | 490  (79%) | 112 (18%) | 20  (3%) |
|  | GIA | 23  (12-40, 0.8-94) | 206/705 (29%) | 343/707 (49%) | 1/63 (2%) | 310  (68%) | 118 (26%) | 29  (6%) |
|  | THA | 20  (9-34, 0.6-89) | 154/389 (40%) | 194/389 (50%) | 0/37 (0%) | 182  (57%) | 108 (34%) | 29  (9%) |
|  | All Vietnam | 22  (10-36, 0.1-94) | 1,112/3,194 (35%) | 1,619/3,207 (50%) | 2/184 (1%) | 1,535  (74%) | 445  (21%) | 91  (4%) |
|  | OVERALL | 21  (9-36, 0.1-94) | 2,695/7,333 (37%) | 3,785/7,350 (51%) | 57/741 (8%) | 4,097  (81%) | 815  (16%) | 124  (2%) |

Cam= Cambodia, IQR= inter-quartile range, y.o. = years old, No. = number, g/dL= grams per decilitre *temp>37.5^o^C
