# Supplementary material for: The epidemiology of subclinical malaria infections in South-East Asia: findings from cross-sectional surveys in Thailand–Myanmar border areas, Cambodia, and Vietnam
Source: Malar J. 2015 Sep 30;14:381. doi: 10.1186/s12936-015-0906-x (PMC4590703; doi:10.1186/s12936-015-0906-x)
Supplement: Supplementary file 4 — 10.1186/s12936-015-0906-x-S4.docx The results of RDT, light microscopy, and HVUSqPCR in Cambodia, Thailand Myanmar border areas, and Vietnam (disaggregated by village). [file 12936_2015_906_MOESM4_ESM.docx]

The results of RDT, light microscopy, and HVUSqPCR in Cambodia, Thailand Myanmar border areas, and Vietnam (disaggregated by village)

|  | RDT | | | | | | | | | Microscopy | | | | | | | HVUSqPCR | | | | | | | | | | |
| --- | --- | --- | --- | --- | --- | --- | --- | --- | --- | --- | --- | --- | --- | --- | --- | --- | --- | --- | --- | --- | --- | --- | --- | --- | --- | --- | --- |
| Location | n | No. pos | % | Pf | % | non- PF | % | mixed | % | n | No. pos | % | Pf | % | Pv | % | n | No. pos | % | Pf | % | Pv | % | mixed | % | P. spp. | % |
| KL | 529 | 0 | 0% | 0 | 0% | 0 | 0% | 0 | 0% | 529 | 2 | 0% | 0 | 0% | 2 | 0% | 529 | 100 | 19% | 21 | 4% | 25 | 5% | 2 | 0% | 52 | 10% |
| OK | 298 | 0 | 0% | 0 | 0% | 0 | 0% | 0 | 0% | 298 | 1 | 0% | 0 | 0% | 1 | 0% | 298 | 22 | 7% | 0 | 0% | 4 | 1% | 0 | 0% | 18 | 6% |
| PDB | 620 | 1 | 0% | 1 | 0% | 0 | 0% | 0 | 0% | 620 | 5 | 1% | 1 | 0% | 4 | 1% | 620 | 107 | 17% | 11 | 2% | 19 | 3% | 2 | 0% | 75 | 12% |
| Cambodia | 1447 | 1 | 0% | 1 | 0% | 0 | 0% | 0 | 0% | 1447 | 8 | 1% | 1 | 0% | 7 | 0% | 1,447 | 229 | 16% | 32 | 2% | 48 | 3% | 4 | 0% | 145 | 10% |
| HKT | 524 | 70 | 13% | 42 | 8% | 28 | 5% | 0 | 0% | 539 | 54 | 10% | 9 | 2% | 45 | 8% | 540 | 156 | 29% | 23 | 4% | 82 | 15% | 9 | 2% | 42 | 8% |
| KNH | 276 | 56 | 20% | 49 | 18% | 7 | 3% | 0 | 0% | 274 | 37 | 14% | 20 | 7% | 17 | 6% | 279 | 105 | 38% | 40 | 14% | 34 | 12% | 10 | 4% | 21 | 8% |
| TOT | 407 | 21 | 5% | 10 | 2% | 11 | 3% | 0 | 0% | 411 | 35 | 9% | 7 | 2% | 28 | 7% | 409 | 187 | 46% | 16 | 4% | 71 | 17% | 1 | 0% | 99 | 24% |
| TPN | 177 | 11 | 6% | 7 | 4% | 4 | 2% | 0 | 0% | 308 | 18 | 6% | 3 | 1% | 15 | 5% | 308 | 72 | 23% | 8 | 3% | 43 | 14% | 1 | 0% | 20 | 6% |
| Thailand Myanmar border | 1384 | 158 | 11% | 108 | 8% | 50 | 4% | 0 | 0% | 1532 | 144 | 9% | 39 | 3% | 105 | 7% | 1,536 | 520 | 34% | 87 | 6% | 230 | 15% | 21 | 1% | 182 | 12% |
| BB | 692 | 19 | 3% | 11 | 2% | 6 | 1% | 2 | 0% | 652 | 18 | 3% | 6 | 1% | 12 | 2% | 659 | 92 | 14% | 14 | 2% | 33 | 5% | 7 | 1% | 38 | 6% |
| BK | 618 | 26 | 4% | 21 | 3% | 5 | 1% | 0 | 0% | 631 | 9 | 2% | 5 | 1% | 4 | 1% | 554 | 41 | 7% | 5 | 1% | 15 | 3% | 3 | 1% | 18 | 3% |
| GIA | 539 | 11 | 2% | 3 | 1% | 5 | 1% | 3 | 1% | 527 | 31 | 6% | 8 | 2% | 21 | 4% | 484 | 71 | 14% | 13 | 3% | 18 | 4% | 16 | 3% | 24 | 5% |
| THA | 328 | 9 | 3% | 4 | 1% | 2 | 1% | 3 | 1% | 322 | 19 | 7% | 8 | 2% | 9 | 3% | 295 | 35 | 12% | 13 | 4% | 13 | 4% | 5 | 2% | 4 | 1% |
| Vietnam | 2177 | 65 | 3% | 39 | 2% | 18 | 1% | 8 | 0% | 2132 | 77 | 4% | 27 | 1% | 46 | 2% | 1,992 | 239 | 12% | 45 | 2% | 79 | 4% | 31 | 2% | 84 | 4% |
| Overall | 5008 | 224 | 4% | 148 | 3% | 68 | 1% | 8 | 0% | 5111 | 229 | 5% | 67 | 1% | 158 | 3% | 4,975 | 988 | 20% | 164 | 3% | 357 | 7% | 56 | 1% | 411 | 8% |

RDT=rapid diagnostic test, HVUSqPCR= high volume ultra-sensitive real time polymerase chain reaction

n= number of participants, No. pos=number of participants with a positive test result, Pf= *Plasmodium falciparum*, non-Pf= RDTs distinguish between *P. falciparum*, non-*P. falciparum*, and no infections, mixed= more than one *Plasmodium* species was identified Pv= *Plasmodium vivax*, P.spp.= *Plasmodium* species not identified

Light microscopy did not detect mixed infections
