# Supplementary material for: The epidemiology of subclinical malaria infections in South-East Asia: findings from cross-sectional surveys in Thailand–Myanmar border areas, Cambodia, and Vietnam
Source: Malar J. 2015 Sep 30;14:381. doi: 10.1186/s12936-015-0906-x (PMC4590703; doi:10.1186/s12936-015-0906-x)
Supplement: Supplementary file 5 — 10.1186/s12936-015-0906-x-S5.docx Sensitivity, specificity, positive predictive value and negative predictive value of RDTs and microscopy compared with HVUSqPCR species detection (disaggregated by village). [file 12936_2015_906_MOESM5_ESM.docx]

Sensitivity, specificity, positive predictive value and negative predictive value of RDTs and microscopycompared with HVUSqPCR species detection (disaggregated by village)

|  |  | RDT | | | | | | | | | microscopy | | | | | | | | |
| --- | --- | --- | --- | --- | --- | --- | --- | --- | --- | --- | --- | --- | --- | --- | --- | --- | --- | --- | --- |
|  |  | qPCR  pos RDT pos | qPCR pos  RDT  neg | qPCR neg RDT neg | qPCR neg RDT pos | Total | Sensitivity  (95% C.I.) | Specificity  (95% C.I.) | positive predictive value  (95% C.I.) | negative predictive value  (95% C.I.) | qPCR  pos micro pos | qPCR pos  micro neg | qPCR neg micro neg | qPCR neg micro pos | Total | Sensitivity  (95% C.I.) | Specificity  (95% C.I.) | positive predictive value  (95% C.I.) | negative predictive value  (95% C.I.) |
| Pf | All sites | 96 | 120 | 4462 | 55 | 4733 | 44%  (38 to 51%) | 99%  (98 to 99%) | 64%  (55 to 71%) | 97%  (97 to 98%) | 66 | 144 | 4616 | 5 | 4827 | 33%  (24 to 36%) | 100%  (99 to 100%) | 93%  (83 to 98%) | 97%  (96 to 97%) |
|  | Cam | 1 | 35 | 1411 | 0 | 1447 | 3%  (0 to 15%) | 100%  (99 to 100%) | 100%  (3 to 100%) | 98%  (97 to 98%) | 0 | 36 | 1410 | 1 | 1447 | 0%  (0 to 10%) | 100%  (99 to 100%) | 0%  (0 to 10%) | 98%  (97 to 98%) |
|  | TMBA | 71 | 34 | 1241 | 37 | 1383 | 68%  (58 to 76%) | 97%  (96 to 98%) | 66%  (56 to 75%) | 97%  (96 to 98%) | 37 | 61 | 1418 | 1 | 1517 | 38%  (28 to 48%) | 100%  (99 to 100%) | 98%  (97 to 98%) | 96%  (95 to 97%) |
|  | Viet | 24 | 51 | 1810 | 18 | 1903 | 32%  (22 to 44%) | 99%  (98 to 100%) | 57%  (41 to 72%) | 97%  (96 to 98%) | 25 | 47 | 1788 | 3 | 1863 | 38%  (24 to 47%) | 100%  (99 to 100%) | 89%  (72 to 98%) | 97%  (97 to 98%) |
| non-PF | All sites | 54 | 332 | 4332 | 15 | 4733 | 14%  (11 to 18%) | 100%  (99 to 100%) | 78%  (67 to 87%) | 93%  (92 to 94%) | 141 | 262 | 4411 | 13 | 4827 | 35%  (30 to 40%) | 100%  (99 to 100%) | 92%  (86 to 95%) | 94%  (94 to 95%) |
|  | Cam | 0 | 52 | 1395 | 0 | 1447 | 0%  (0 to 1%) | 100%  (93 to 100%) | na | 96%  (95 to 97%) | 7 | 45 | 1395 | 0 | 1447 | 14%  (6 to 26%) | 100  (99 to 100%) | 100  (59 to 100%) | 97%  (96 to 98%) |
|  | TMBA | 47 | 180 | 1153 | 3 | 1383 | 21%  (16 to 27%) | 100%  (99 to 100%) | 94%  (84 to 99%) | 87%  (85 to 88%) | 99 | 149 | 1264 | 5 | 1517 | 40%  (34 to 46%) | 100%  (99 to 100%) | 95%  (89 to 98% | 97%  (96 to 98%) |
|  | Viet | 7 | 100 | 1784 | 12 | 1903 | 7%  (3 to 13%) | 100%  (99 to 100%) | 37%  (16 to 62%) | 95%  (94 to 96%) | 35 | 68 | 1752 | 8 | 1863 | 34  (25 to 44%) | 100%  (99 to 100%) | 81%  (67 to 92%) | 96%  (95 to 97%) |
| P. spp. | All sites | 20 | 379 | 3740 | 46 | 4185 | 5%  (3 to 8%) | 99%  (98 to 99%) | 30%  (20 to 43%) | 91%  (90 to 92% | 13 | 391 | 3864 | 1 | 4269 | 3%  (2 to 5%) | 100%  (99 to 100%) | 93%  (66 to 100%) | 91%  (90 to 92%) |
|  | Cam | 0 | 145 | 1218 | 0 | 1363 | 0%  (0 to 0.3%) | 100%  (97 to 100%) | na | 89%  (88 to 91%) | 0 | 145 | 1218 | 0 | 1363 | 0%  (0 to 0.3%) | 100%  (97 to 100%) | na | 89%  (88 to 91%) |
|  | TMBA | 16 | 158 | 873 | 24 | 1071 | 9%  (5 to 15%) | 97%  (96 to 98%) | 40%  (25 to 57%) | 85%  (82 to 87%) | 6 | 175 | 1009 | 0 | 1190 | 3%  (1 to 7%) | 100%  (99 to 100%) | 100%  (54 to 100%) | 85%  (83 to 87%) |
|  | Viet | 4 | 76 | 1649 | 22 | 1751 | 5%  (1 to 12%) | 99%  (98 to 99%) | 15%  (4 to 35%) | 96%  (95 to 97%) | 7 | 71 | 1637 | 1 | 1716 | 9%  (4 to 18%) | 100%  (99 to 100%) | 88%  (47 to 100%) | 96%  (95 to 97%) |

RDT=rapid diagnostic test, HVUSqPCR= high volume ultra-sensitive real time polymerase chain reaction

n= number of participants, No. pos=number of participants with a positive test result, Pf= *Plasmodium falciparum*, non-Pf= RDTs distinguish between P. falciparum, non-P.falciparum, and no infections, mixed= more than one *Plasmodium* species was identified Pv= *Plasmodium vivax*, P.spp. = *Plasmodium* species not identified

Light microscopy did not detect mixed infections
