# Supplementary figures and images for: The epidemiology of subclinical malaria infections in South-East Asia: findings from cross-sectional surveys in Thailand–Myanmar border areas, Cambodia, and Vietnam
Source: Malar J. 2015 Sep 30;14:381. doi: 10.1186/s12936-015-0906-x (PMC4590703; doi:10.1186/s12936-015-0906-x)

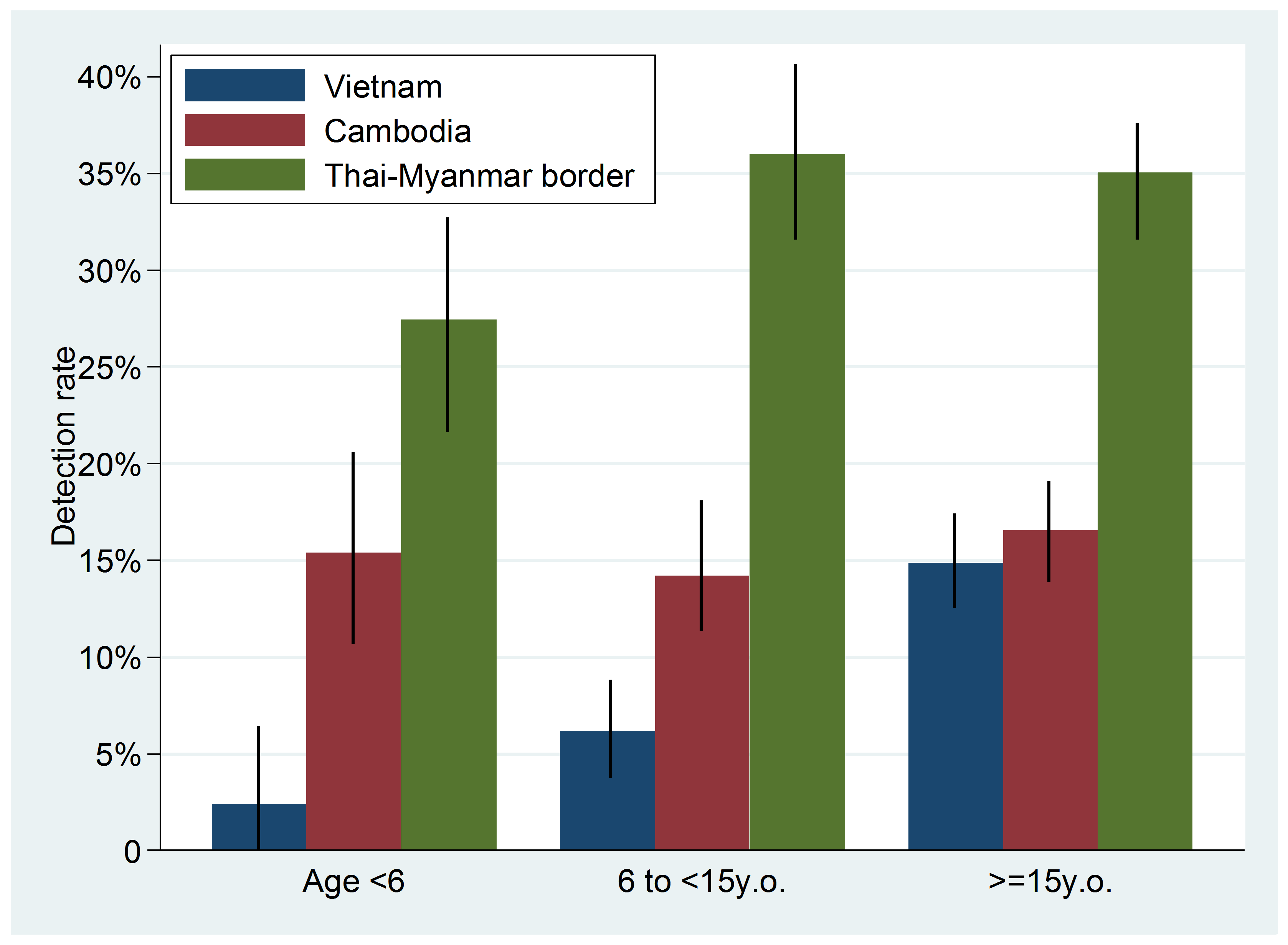

Supplement: Supplementary file 6 — 10.1186/s12936-015-0906-x-S6.tif Parasite prevalence detected by HVUSqPCR by age group. [file 12936_2015_906_MOESM6_ESM.tif]
