# Supplementary material for: The epidemiology of subclinical malaria infections in South-East Asia: findings from cross-sectional surveys in Thailand–Myanmar border areas, Cambodia, and Vietnam
Source: Malar J. 2015 Sep 30;14:381. doi: 10.1186/s12936-015-0906-x (PMC4590703; doi:10.1186/s12936-015-0906-x)
Supplement: Supplementary file 7 — 10.1186/s12936-015-0906-x-S7.docx Adjusted odds for being HVUSqPCR positive stratified by household, random effects modelling by country. In Cambodia the initial model included resident (0/1), anemia (mild, moderate, none), fever (defined as a temperature >37.5 °C) at presentation* (0/1), history of fever (0/1), sex, age group (<6 year-old, ≥6 year-old–<15 year-old, and ≥15 year-old), height, weight, history of illness (0/1), history of malaria (0/1), previous anti-malarials (0/1), use of bednets (0/1), recent visit to forest (0/1), occupation (0/1), family (0/1) and village. In TMBA the initial model included anemia (mild, moderate, none), fever at presentation (0/1), history of fever (0/1), sex, age group <6 year-old, ≥6 year-old–<15 year-old, and ≥15 year-old), occupation (0/1), family (0/1) and village. In Vietnam the initial model included resident (0/1), anemia (mild, moderate, none), fever at presentation* (0/1), history of fever (0/1), sex, age group (<6 year-old, ≥6 year-old–<15 year-old, and ≥15 year-old), height, weight, history of illness (0/1), history of malaria (0/1), use of bednets (0/1), recent visit to forest (0/1), occupation (0/1) and village. [file 12936_2015_906_MOESM7_ESM.docx]

**Adjusted odds for being HVUSqPCR positive stratified by household, random effects modelling by country.**

In Cambodia the initial model included resident (0/1), anemia (mild, moderate, none), fever (defined as a temperature > 37.5°C) at presentation* (0/1), history of fever (0/1), sex, age group (<6 y.o., ≥6 y.o. to <15 y.o., and ≥15 y.o.), height, weight, history of illness (0/1), history of malaria (0/1), previous anti-malarials (0/1), use of bednets (0/1), recent visit to forest (0/1), occupation (0/1), family (0/1) and village. In TMBA the initial model included anemia (mild, moderate, none), fever at presentation (0/1), history of fever (0/1), sex, age group (<6 y.o., ≥6 y.o. to <15 y.o., and ≥15 y.o.), occupation (0/1), family (0/1) and village. In Vietnam the initial model included resident (0/1), anemia (mild, moderate, none), fever at presentation* (0/1), history of fever (0/1), sex, age group (<6 y.o., ≥6 y.o. to <15 y.o., and ≥15 y.o.), height, weight, history of illness (0/1), history of malaria (0/1), use of bednets (0/1), recent visit to forest (0/1), occupation (0/1) and village.

|  | Factor | No. qPCR positive with factor (%) | No. qPCR positive without factor (%) | OR | (95% CI) |
| --- | --- | --- | --- | --- | --- |
| Cambodia |  |  |  |  |  |
|  | Not-resident in OK^a^ | 22/298 (7%) | ^a^ | 3.13 | (1.75 to 5.56) |
|  | History of fever^b^ | 43/182 (24%) | 185/1263 (15%) | 2.01 | (1.31 to 3.08) |
|  | History of malaria | 113/536 (21%) | 115/909 (13%) | 1.79 | (1.31 to 2.46) |
|  | Male | 131/728 (18%) | 97/717 (14%) | 1.40 | (1.03 to 1.91) |
| TMBA |  |  |  |  |  |
|  | Male | 299/779 (38%) | 214/739 (28.96) | 1.74 | (1.37 to 2.21) |
|  | Village ^c^ |  |  |  |  |
|  | KNH | 105/277 (38%) | ^c^ | 1.53 | (1.03 to 2.27) |
|  | TOT | 182/404 (45%) | ^c^ | 2.44 | (1.71 to 3.49) |
|  | Age 6 to 15 ^d^ | 129/361 (36%) | ^d^ | 1.96 | (1.31 to 2.93) |
|  | Age 15+ ^d^ | 312/891 (35%) | ^d^ | 2.03 | (1.41 to 2.91) |
|  | Mild anaemia ^e^ | 69/182 (38%) | ^e^ | 1.51 | (1.04 to 2.21) |
|  | Moderate anaemia ^e^ | 9/18 (50%) | ^e^ | 2.74 | (0.96 to 7.86) |
| Vietnam |  |  |  |  |  |
|  | History of malaria | 56/247 (23%) | 97/1058 (9%) | 2.11 | (1.36 to 3.27) |
|  | Height | - | - | 1.03 | (1.02 to 1.05) |
|  | Resident in village BK ^f^ | 28/407 (7%) | ^f^ | 0.43 | (0.25 to 0.74) |
|  | Use bednet | 139/1268 (11%) | 14/37 (38%) | 0.31 | (0.14 to 0.74) |
|  | Recent visit to forest | 83/340 (24%) | 70/965 (7%) | 2.49 | (1.66 to 3.73) |
|  | Male | 96/592 (16%) | 57/713 (8%) | 1.63 | (1.08 to 2.44) |

^a^ compared against KL (100/529 (19%)); village PDB not significant (106/618 (17%)).^b^ temperature > 37.5°C; ^d^ compared against age <6 (72/266 (27%)); ^c^ compared against HKT (154/530 (29%)); village TPN not significant (72/307 (24%)); ^e^ compared against no anemia (435/1318 (33%)); ^f^ compared against BB (64/469 (14%)); village GIA and THA not significant (50/330 (15%) and 11/99 (11%), respectively).
